# Adsorption Properties of Nano-MnO_2_–Biochar Composites for Copper in Aqueous Solution

**DOI:** 10.3390/molecules22010173

**Published:** 2017-01-20

**Authors:** Li Zhou, Yifan Huang, Weiwen Qiu, Zhanxiang Sun, Zhongqi Liu, Zhengguo Song

**Affiliations:** 1 College of Land and Environment, Shenyang Agricultural University, Shenyang 110866, China; lily_621@163.com; 2 Agro-Environmental Protection Institute, Ministry of Agriculture of China, Key Laboratory of Production Environment Quality, Ministry of Agriculture of China, Tianjin 300191, China; yifan_huang@outlook.com (Y.H.); liuzhongqi508@163.com (Z.L.); 3 The New Zealand Institute for Plant and Food Research Limited, Private Bag 4704, Christchurch 8140, New Zealand; weiwen.qiu@plantandfood.co.nz; 4 Liaoning Academy of Agriculture Science, Shenyang 110866, China; song19961101@tom.com

**Keywords:** biochar, nano-MnO_2_–biochar composites, adsorption, copper

## Abstract

There is a continuing need to develop effective materials for the environmental remediation of copper-contaminated sites. Nano-MnO_2_–biochar composites (NMBCs) were successfully synthesized through the reduction of potassium permanganate by ethanol in a biochar suspension. The physicochemical properties and morphology of NMBCs were examined, and the Cu(II) adsorption properties of this material were determined using various adsorption isotherms and kinetic models. The adsorption capacity of NMBCs for Cu(II), which was enhanced by increasing the pH from 3 to 6, was much larger than that of biochar or nano-MnO_2_. The maximum adsorption capacity of NMBCs for Cu(II) was 142.02 mg/g, which was considerably greater than the maximum adsorption capacities of biochar (26.88 mg/g) and nano-MnO_2_ (93.91 mg/g). The sorption process for Cu(II) on NMBCs fitted very well to a pseudo-second-order model (*R*^2^ > 0.99). Moreover, this process was endothermic, spontaneous, and hardly influenced by ionic strength. The mechanism of Cu(II) adsorption on NMBCs mainly involves the formation of complexes between Cu(II) and O-containing groups (e.g., COO–Cu and Mn–O–Cu). Thus, NMBCs may serve as effective adsorbents for various environmental applications, such as wastewater treatment or the remediation of copper-contaminated soils.

## 1. Introduction

The continual entry of copper into the environment, owing to long-term anthropogenic activities, such as mining, smelting, metal processing, and the application of copper salts as fungicides, has seriously impacted soil and water safety and increased the exposure risk to humans [1]. Excess copper can deposit in the human brain, liver, pancreas, and myocardium, causing liver disease and Wilson’s disease [2,3]. Frequently used methods for copper removal include chemical precipitation [4], coprecipitation/sorption with hydrous metal oxides such as manganese, filtration, and adsorption [5]. Of these methods, adsorption is a highly effective [6] and attractive process because it is simple and cost efficient [7]. Several types of adsorbents have been applied for the removal of copper from wastewater. Since carbonaceous materials have relatively large specific areas and high adsorption capacities, they are more commonly used as adsorbents than metal oxides, clays, etc. [8,9].

Recently, biochar has been widely used for heavy metal removal from aqueous solutions because it is cheap, abundant, and readily available. Biochar is a carbon-rich material derived from the thermal decomposition of biomass in a closed system with little or no oxygen [10,11]. Some recent studies showed that biochar is an effective sorbent for the removal of heavy metals, including lead, copper, nickel, and cadmium, from aqueous solutions [8,9,12]. In particular, the adsorption capacity of biochar for Cu is 5–80 mg/g [13,14]. In an effort to increase the adsorption capacity of biochar for heavy metals, several studies have reported that the ability of biochar and other carbon materials to sorb heavy metals is enhanced by modification with metallic oxides or inorganic minerals [1,15,16]. Uchimiya et al. [17] showed that biochar derived from cottonseed hulls contains considerably more carboxyl functional groups and has a significantly greater ability to stabilize Pb, Cu, and Zn after treatment with H_2_SO_4_/HNO_3_ than does unoxidized biochar.

High-surface-area manganese oxides are effective sorbents for the removal of heavy metals from aqueous solutions because of their affinity for several heavy metals [18,19]. Moreover, nano-MnO_2_ has a large number of reactive hydroxyl groups on the surface, which results in large adsorption capacities for heavy metal ions [20]. Although the adsorption capacity of nano-MnO_2_ for copper is very good (up to 100 mg/g), these materials are expensive and aggregate easily in aqueous solution, which limits their practical applications. In recent years, these drawbacks have led to a growing interest in the synthesis of novel adsorbents by loading an oxide onto another solid material. Several adsorbents, including manganese dioxide-coated sand [21], manganese dioxide-loaded resin [22], and cellulose-loaded nanoscale-manganese oxide [23], have been reported. Despite the high adsorption capacities shown by these sorbents, they are not cost effective because the production processes are expensive and the post-treatment procedures may not be environmentally friendly.

In a previous study, we synthesized a MnO_x_–biochar composite, which was confirmed to have good adsorption properties for Cu(II) [1]. In the current study, to reduce energy consumption and cost, a simple precipitation technique was employed to prepare the homogeneous nano-MnO_2_–biochar composites (NMBCs) at room temperature. The objectives of this study were (1) to evaluate the surface physicochemical properties of NMBCs; (2) to examine the sorption of Cu(II) on NMBCs; and (3) to investigate the mechanism of Cu(II) sorption on NMBCs.

## 2. Results and Discussion

### 2.1. Characterization of Samples 

In this study, NMBCs were prepared by modifying biochar (BC) samples with nanostructured manganese oxides (NMnO_2_). As nanoparticles can readily aggregate in aqueous solution, it is difficult to obtain a homogeneous mixture of NMnO_2_ and BC in water. In this study, NMnO_2_ was deposited directly onto the surface of BC, which impaired the aggregation of nanoparticles. The manganese dioxide on the surface of NMBCs is likely to grow as a matrix structure that is much more homogeneous than that obtained in a physical mixture of NMnO_2_ and BC. Selected physicochemical properties of BC, NMnO_2_, and NMBCs are presented in Table 1. The relative elemental contents of C and H in BC dramatically decreased after NMnO_2_ modification. In contrast, the bulk content of O increased from 5.16% in BC to 17% in NMBCs, indicating that NMnO_2_ modification greatly increased the O-containing groups in NMBCs. The content of Mn in NMBCs, determined by digestion (75.6 mg/g), was comparable to the value calculated based on the weight ratio of NMnO_2_ to BC (93.8 mg/g). As the surface area of NMBCs (80.3 m^2^/g) is much larger than that of BC (61 m^2^/g), NMBCs can provide more sites for Cu(II) adsorption. Compared with BC, the surface of NMBCs has more porous structures, indicated by the similar decreases observed in pore size and volume. The pore volume of the NMBCs was about 3 times smaller than that of the BC and 1.5 times smaller than that of NMnO_2_. The average pore diameter of the NMBCs was 3.8 nm, which is large enough to accommodate Cu(II) (hydrated radius: 41.9 Å). Similarly, the passage of ions through a nanofiltration membrane has strong correlation with their hydrated radii [24].

**Figure 1:**
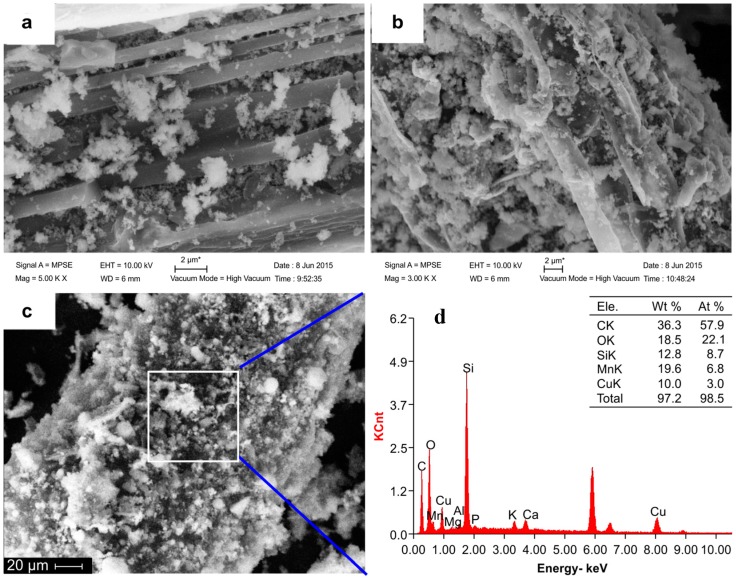
SEM images of the nano-MnO_2_–biochar composites (NMBCs) (**a**) NMBCs before the adsorption of Cu; (**b**) the NMBCs after adsorption of Cu; (**c**) SEM-EDS image of the NMBCs after the adsorption of Cu; and (**d**) the EDS test result after the adsorption of Cu.

To further examine the properties of NMBCs, scanning electron microscopy (SEM) images were obtained (Figure 1a,b). The surface of the NMBCs was covered with nano-MnO_2_, which formed various porous structures. The MnO_2_ nanospheres were densely grown on the entire surface of the biochar, and some of the nanospheres were aggregated, with diameters of about 30 nm (Figure 1a,b). The formation of microspherical structures on the surface of the biochar greatly enlarged the surface area of the sample. Following the exposure of NMBCs to an aqueous solution of Cu(II), SEM-EDS analysis showed that Cu(II) was adsorbed on the surface of the NMBCs (Figure 1c), indicating that NMBCs have an adsorption capacity for Cu(II).

### 2.2. Adsorption Kinetics

**Figure 2:**
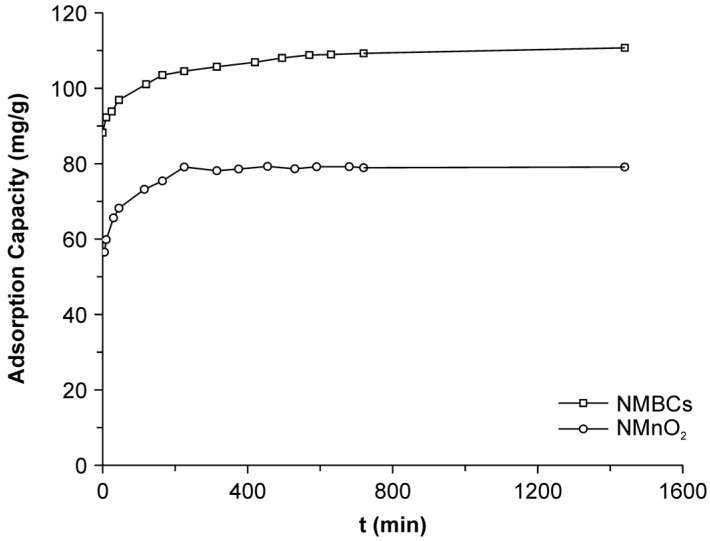
Adsorption kinetics of Cu(II) by NMBCs and NMnO_2_.

The sorption kinetics of Cu(II) onto NMnO_2_ and NMBCs are shown in Figure 2. The adsorption of Cu(II) by NMBCs increases quickly within the first 200 min, reaching equilibrium after 600 min. The data indicate that the Cu(II) sorption process could be divided into rapid and slow stages. The rapid sorption phase may be ascribed to the rapid occupation of easily accessible external surface sorption sites, likely via physical sorption.

To understand the dynamics of the adsorption process, the kinetic data were analyzed using pseudo-first-order and pseudo-second-order models. The pseudo-second-order equation is based on the assumption that the sorption rate is controlled by both the sorbent capacity and the sorbate concentration and is expressed as:

(1) $$\frac{t}{q_{t}}=\frac{1}{{q_{e}}^{2}k_{2}}+\frac{t}{q_{e}},$$

where *K*_2_ is the pseudo-second-order rate constant (g/mg·h) and *q_e_* and *q_t_* represent the amount of Cu(II) sorbed (mg/g) at equilibrium and at time *t*, respectively. The fit of the data to a pseudo-second-order model was better than that to a pseudo-first-order model (*R*^2^ = 0.99 and *R*^2^ = 0.52, respectively). The obtained parameters for the pseudo-second-order kinetic adsorption model are shown in Table 2. The equilibrium sorption capacity (*q*_e_ = 110 mg/g) and the second-order rate constant (*K*_2_ = 12.26 g/mg·h) of the NMBCs were determined from the slope and intercept of the plot of *t/q* versus *t* (data not shown). The good fit of the data to this model showed that the removal of Cu(II) by NMBCs proceeds via a chemisorption process.

### 2.3. Adsorption Isotherms 

**Figure 3:**
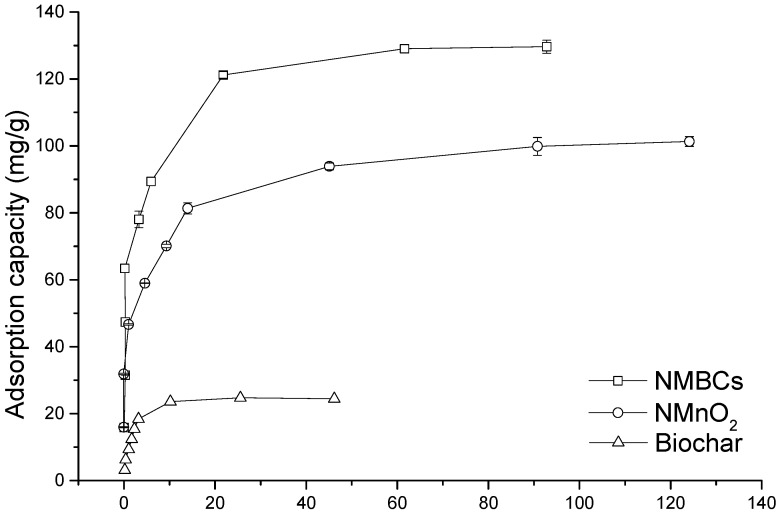
Adsorption isotherms of NMBCs, NMnO_2_, and BC.

An adsorption isotherm indicates how molecules are distributed between the liquid and solid phases when the adsorption process reaches an equilibrium state. The Cu(II) adsorption isotherms of BC, NMnO_2_, and NMBCs are shown in Figure 3. The adsorption capacity of NMBCs is significantly higher than that of BC or NMnO_2_.

There are several isotherm equations available for analyzing experimental adsorption equilibrium data. The Langmuir isotherm is based on monolayer adsorption (constant heat of adsorption for all sites) at finite homogeneous sites within an adsorbent. The Freundlich isotherm is derived by assuming a heterogeneous surface (multilayer adsorption) with a non-uniform distribution of the heat of adsorption over the surface. This isotherm assumes that the adsorption sites are distributed exponentially, with respect to the heat of adsorption, and can be used to describe chemisorption processes. In this study, both the Langmuir and Freundlich models were used to fit the data for adsorption of heavy metal ions by NMBCs [25].

(2) $$\mathrm{Langmuir}\;\mathrm{equation}:q_{e}=\frac{q_{m}bC_{e}}{1+bC_{e}},$$

(3) Langmuir equation:qe=KFCe1n,

In the Langmuir equation, *q_e_* is the equilibrium adsorption capacity of the adsorbent (mg/g) and *C_e_* is the equilibrium concentration (mg/L); the other parameters are various isotherm constants that are determined by nonlinear regression of the experimental data. In the Freundlich equation, *q*_e_ is the amount of copper adsorbed per unit of mass of the adsorbent at equilibrium (mg/g), *C*_e_ is the equilibrium copper concentration (mg/L), and *K_F_* and *n* are parameters related to the adsorption capacity and the intensity of adsorption, respectively.

The estimated model parameters and corresponding correlation coefficients are summarized in Table 3. Both the Langmuir and Freundlich models fitted the data reasonably well. According to the Langmuir model, the maximum adsorption capacity of the NMBCs was 140.02 mg/g, whereas those of BC and NMnO_2_ were 26.88 and 93.91 mg/g, respectively, showing the greater Cu(II) adsorption capacity of NMBCs. Some previous studies have reported that the Cu(II) adsorption capacities of BC, modified BC, and modified graphene are in the range of several mg/g to ~160 mg/g [1,8,26,27]. The NMBCs prepared in the current study show similarly high adsorption capacities, indicating that loading NMnO_2_ onto BC greatly enhances the adsorption of Cu(II). Moreover, NMBCs are much cheaper and easier to obtain in large quantities than are graphene sheets and iron or aluminum oxide-modified BC. Thus, NMBCs may be an excellent candidate for the effective removal of copper from aqueous environments.

### 2.4. Adsorption Thermodynamics

**Figure 4:**
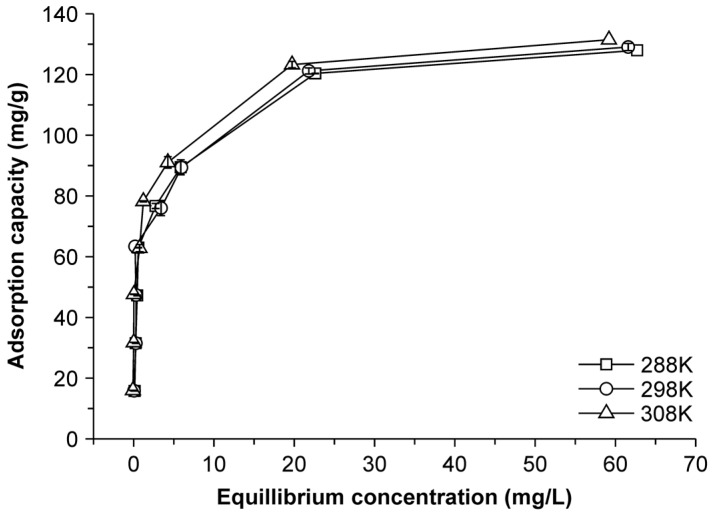
Effect of temperature on the Cu(II) adsorption properties of NMBCs.

To understand the effect of temperature on Cu(II) adsorption by NMBCs (Figure 4), thermodynamic parameters, such as the changes in the standard free energy (Δ*G*°, J/mol), enthalpy (Δ*H*°, J/mol), and entropy (Δ*S*°, J/K·mol), were calculated using the following thermodynamic relations [28]:

Δ*G*° = Δ*H*° − *T*Δ*S*°, (4)

Δ*G*° = −2.303*RT* log *K_e_* (5)

where *T* (K) is the absolute temperature, *K_e_* is the equilibrium constant, and *R* (8.314 J/K·mol) is the universal gas constant.

By plotting ln *Ke* against *1/T*, the values of Δ*H*° and Δ*S*° can be estimated from the slopes and intercepts, and the values of Δ*G*° can be obtained from the corresponding values of Δ*H*° and Δ*S*° (plots not shown). Δ*G*° at various temperatures was calculated using Equation (5), and the calculated thermodynamic parameters are listed in Table 4. The positive values of Δ*H*° suggest that the adsorption of Cu(II) by NMBCs is endothermic. An increase in the entropy accompanies the adsorption reaction, as shown by the positive values of Δ*S*°, whereas the negative values of Δ*G*° indicate that the adsorption process is spontaneous in nature [29].

### 2.5. Effect of pH on Adsorption

**Figure 5:**
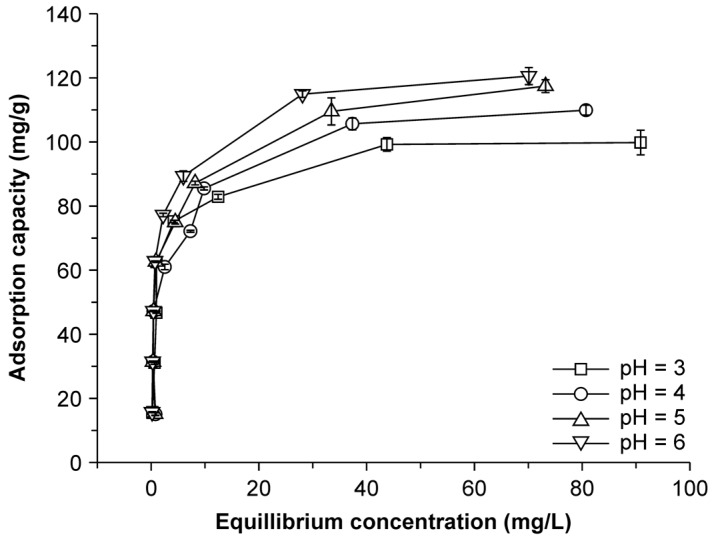
Effect of pH on the Cu(II) adsorption properties of NMBCs.

The adsorption capacity of the NMBCs showed a distinct increase on increasing the pH from 3 to 6 (Figure 5). Cu is also affected by pH; above a critical pH in an aqueous solution, the formation of Cu hydroxides or the precipitation of Cu may occur. As these experiments were performed at pH values in the range of 3–6, the Cu in the aqueous solution can be regarded as completely in the ionic state. The pH can affect the adsorption capacity in two ways; first, the surface electric charge density can facilitate or reduce electrostatic interactions, and, second, ion exchange and metal ion deposition can affect the metal species in an aqueous solution [30]. The surface of NMBCs is positively charged when the pH of the solution (pH 3–6) is lower than the point of zero charge (pH_ZPC_) of NMBCs (11.0). The adsorption of metal ions by NMBCs is weaker at lower pH values than at higher pH values because the high concentrations of H^+^ and H_3_O^+^ effectively compete with metal ions in the system [31]. In NMBCs, NMnO_2_ is loaded onto BC, which greatly increases the available adsorption sites (Figure 1a,b). Increased positive charges in the system increase electrostatic repulsions between cations and weaken the adsorption capacity of NMBCs for both H^+^ and Cu(II). Due to protonation and deprotonation reactions, changes in the pH affect the surface charges. When the pH is low, the surface of NMBCs is protonated, which reduces the adsorption capacity of this material, but in the pH range between 3 and 6, the NMBCs showed significant adsorption capacity for Cu(II), with the maximum Cu(II) adsorption capacity observed at a pH of ~6 (142 mg/g). Therefore, it could be speculated that metal deposition, instead of electrostatic attraction at cation exchange sites, may play an important role in Cu(II) adsorption on NMBCs.

### 2.6. Effect of Ionic Strength on Adsorption

**Figure 6:**
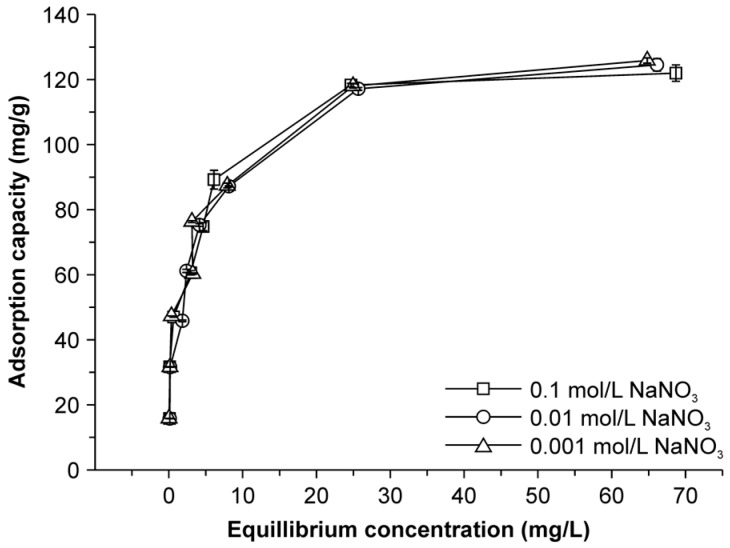
Effect of ionic strength on Cu(II) adsorption by NMBCs.

The adsorption of Cu(II) by BC is most likely affected by the density of negative surface charges and the surface alkalinity. Negative charges on the surface of modified BC result in electrostatic attraction between the active sites and positively charged metal ions. Screening of this interaction could affect Cu(II) precipitation on the surface of the adsorbent. Some reports have shown that the uptake capacity of adsorbents for heavy metals decreases with increasing ionic strength [23,32]. However, the Cu(II) adsorption capacity of the NMBCs was not suppressed by an increase in the concentration of NaNO_3_ (Figure 6). This result reveals that the Cu(II) uptake capacity of the NMBCs was not significantly affected by the ionic strength. Thus, Na^+^ does not compete with Cu(II) for the negatively charged sites, and an elevated Na^+^ concentration does not hinder Cu(II) loading onto the surface sites through electrostatic repulsion. Thus, competitive adsorption cannot be the main mechanism of Cu(II) adsorption by NMBCs.

### 2.7. XPS Analysis

**Figure 7:**
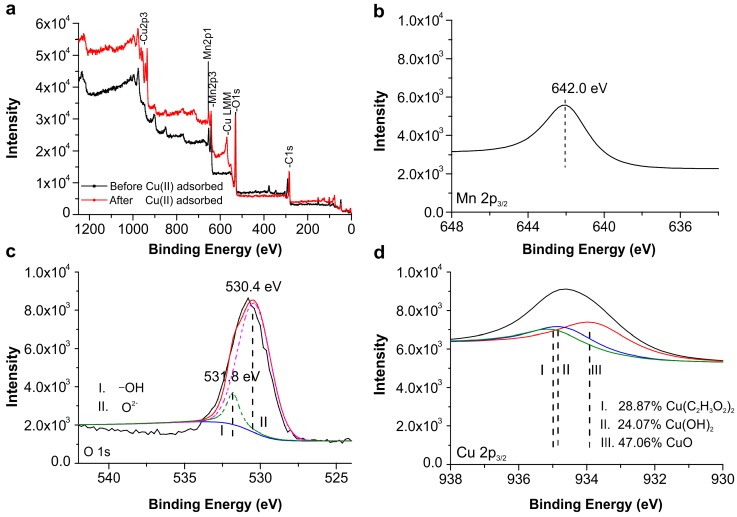
XPS spectra of NMBCs (**a**) survey spectra of NMBCs before and after Cu(II) adsorption; (**b**) Mn 2p_3/2_; (**c**) O 1s spectra of NMBCs before Cu(II) adsorption, the blue line is the baseline and red line is total O 1s; and (**d**) Cu 2p_3/2_ spectrum of NMBCs after Cu(II) adsorption.

X-ray photoelectron spectroscopy (XPS) was performed to probe the bonding interactions in NMBCs before and after adsorption (Figure 7a). The XPS spectrum (Figure 7b) for NMBCs after Cu(II) adsorption contains a Mn 2p_3/2_ peak at about 642.0 eV, with a spin energy separation of 11.6 eV, indicating that the predominant Mn oxidation state has a valence of +4 [33]. The O 1s peak at a binding energy of ~530.4 eV (Figure 7c) corresponds to the lattice oxygens in the form of O^2−^ (metal–oxygen bond), which are increased by loading NMnO_2_ on BC. Another O 1s peak at 531.8 eV was assigned to the surface-adsorbed O in the form of –OH [34]. After reacting with copper, the XPS spectrum of the sample (Figure 7d) showed peaks with binding energies, corresponding to Cu 2p_3/2_. The binding energy of the Cu 2p_3/2_ peak at 934.7 eV indicated the presence of Cu(II). The deconvolution of this peak indicates that the majority of Cu(II) on the surface of NMBCs is in the form of CuO (47.06%), Cu(C_2_H_3_O_2_)_2_ (28.87%), and Cu(OH)_2_ (24.07%). This result clearly indicates that the deposition and chelation of Cu(II) play an important role in the adsorption process.

### 2.8. FTIR Analysis

**Figure 8:**
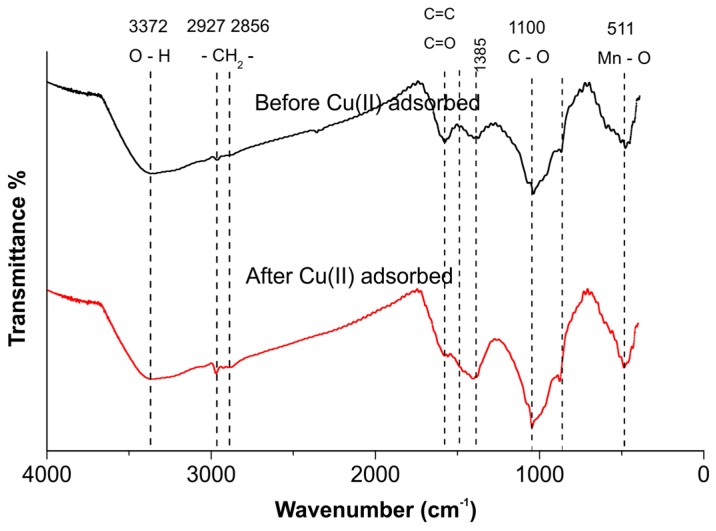
FTIR spectra of NMBCs before and after Cu(II) adsorption.

To elucidate the adsorption mechanism, Fourier transform infrared (FTIR) spectra were recorded to identify the functional groups that affect the adsorption capacity. The FTIR spectra of the NMBCs before and after Cu(II) adsorption are presented in Figure 8. The broad band at ~3369 cm^−1^ is attributed to –OH groups, and the more prominent peak at 1385 cm^−1^ is associated with –OH vibrations [35]. The peaks at 2927 and 2856 cm^−1^ correspond to CH_2_ deformation vibrations [36]. The region from 1745 to 1504 cm^−1^ is ascribed to aliphatic or aromatic groups, such as C=C, and carbonyl C=O stretching vibrations. The broad band observed in the composite in the low-frequency region around 511 cm^−1^ corresponds to Mn–O vibrations [37]. After Cu(II) adsorption, the characteristic bands at 1504, 1385, 1045, and 511 cm^−1^ shifted to lower wavenumbers, which indicates that the peak at 1504 cm^−1^ can be attributed to COO– deformation vibrations and that those at 1385 and 511 cm^−1^ can be attributed to –OH deformation vibrations of hydrated MnO_2_ on the surface of NMBCs. The hydroxyl groups were mostly consumed during the adsorption of Cu(II) to form strong mono- or multidentate inner-sphere complexes (e.g., COO–Cu and Mn–O–Cu), leading to the shift of the characteristic O–H band. The FTIR spectra provided further evidence for strong interactions between Cu(II) and MnO_2_ on the surface of NMBCs. Similarly, the vibrations associated with the hydroxyl groups of graphene nanosheet/MnO_2_ composites and MnO_x_-loaded BC also changed significantly after Cu(II) adsorption [1,27].

## 3. Materials and Methods

### 3.1. Materials

All chemicals were AR grade and purchased from Hengshan Chemical (Tianjin, China). A 1000 mg/L standard solution of copper was obtained from Sigma-Aldrich (St. Louis, MO, USA). The ultrapure water was further purified (18 MΩ·cm) using a Millipore-Q water purification system.

### 3.2. Methods

#### 3.2.1. Preparation of NMBCs

Distilled deionized water (DDW) with a resistivity of 18 MΩ·cm (Millipore, Milford, MA, USA) was used in all procedures. The corn stalk samples were obtained from farmland in a suburb of Tianjin, China. The corn stalks were air-dried at room temperature and milled to pass through a 0.25 mm nylon mesh. The corn stalk powder was placed in corundum crucibles and pyrolyzed at 600 °C for 3 h under flowing N_2_ (1600 mL/min). The obtained char was allowed to cool to room temperature overnight under a N_2_ atmosphere, then ground using a mortar and pestle and sieved using a 150 μm nylon mesh. This biochar sample is referred to as BC.

Nano-MnO_2_ (NMnO_2_) was prepared by adding anhydrous ethanol to an aqueous solution of KMnO_4_ at room temperature. Briefly, 0.5 g of KMnO_4_ was dissolved in 30 mL of DDW in a beaker. Ethanol (10 mL) was added dropwise to the KMnO_4_ solution, which led to the formation of a brownish precipitate of MnO_2_. Details of the formation of NMnO_2_ have been reported by Subramanian et al. [38]. The precipitate was filtered through Whatman No. 42 filter paper and washed extensively with DDW until the pH of the leachate was 6.

The synthesis of the NMBCs was achieved using a simple coprecipitation process at room temperature based on physical interactions between BC and NMnO_2_. First, BC in a beaker was soaked with saturated KMnO_4_ solution and stirred for 1 h with a magnetic stirrer at 25 °C to completely homogenize the mixture. Then, 10 mL of ethanol was added dropwise under constant stirring. A brownish precipitate of NMnO_2_ was observed immediately. The well-mixed precipitate of BC and NMnO_2_ was filtered and dried at 50 °C in a vacuum drying chamber. The obtained NMBCs were ground to pass through a 150 μm nylon sieve. NMBCs contained 70% BC and 30% NMnO_2_.

#### 3.2.2. Sample Characterization 

The nitrogen adsorption isotherms of the BC, NMnO_2_, and NMBCs were measured at 77 K using a NOVA 2000 surface area analyzer (Quantachrome, Boynton Beach, FL, USA). The specific surface area of each adsorbent was determined from the isotherms using the Brunauer–Emmett–Teller (BET) equation. The total amounts of C, H, and N in the BC and NMBCs were determined in triplicate by dry combustion using an Elementar Vario II CHNS/O analyzer (Elementar Analysen systeme GmbH, Hanau, Germany), and the oxygen content was calculated by mass balance. Following wet digestion, atomic absorption spectrometry (AAS; Zeenit 700, Analytik Jena AG, Jena, Germany) at 279.5 nm was used to determine the total amount of Mn in the NMBCs in triplicate.

The surface chemical compositions of the BC, NMnO_2_, and NMBCs were determined using XPS (PHI 5000 Versa Probe, ULVAC-PHI Inc., Chigasaki, Japan). The surface morphologies of the BC, NMnO_2_, and NMBCs were examined using SEM (JEOL, Tokyo, Japan). FTIR spectra were recorded on a Nexus 870 FTIR spectrometer (Nicolet, Madison, WI, USA) in the range of 400–4000 cm^−1^ with a resolution of 4 cm^−1^.

#### 3.2.3. Adsorption Experiments

For the adsorption kinetics studies, 0.5 g of the adsorbent was added to 500 mL of a 127.1 mg/L Cu(II) solution, stirred at 1100 rpm. Samples (1.0 mL) were removed at different time intervals (1, 3, 10, 30, 60, 120, 180, 225, 280, 720, and 1440 min), filtered through a 0.22 μm filter, and then analyzed by AAS.

For the adsorption isotherm studies, a stock solution containing 0.1575 mol/L Cu(NO_3_)_2_ was prepared using Cu(NO_3_)_2_ (guaranteed grade) in DDW. A NaNO_3_ solution (0.1 mol/L) and the appropriate quantities of 0.1575 mol/L Cu(NO_3_)_2_ and DDW were added to 40 mL brown vials containing 100 mg of BC, NMnO_2_, or NMBCs to obtain the final mixed solutions of 0.01 mol/L NaNO_3_, with various concentrations of Cu(NO_3_)_2_ (0.1, 0.25, 0.5, 1.0, 1.5, or 2.0 mmol/L). The final volume was adjusted to 20 mL, and NaNO_3_ (0.01 mol/L) was used to maintain a constant ionic strength during the sorption experiments. The pH values of the suspensions were adjusted to 6.0 by thr dropwise addition of 5.0 mol/L NaOH or 5.0 mol/L HNO_3_.

The effect of pH on adsorption was investigated by adjusting the pH in the range of 3–6, using 0.10 mol/L NaOH and 0.10 mol/L HNO_3_ solutions. The adsorption thermodynamics were examined by varying the temperature (15, 25, and 35 °C). The effect of ionic strength on adsorption was investigated using 0.001, 0.01, and 0.1 mol/L NaNO_3_.

All of the suspensions were agitated using an end-over-end tumbler in a constant-temperature air bath at 25 ± 0.5 °C for 22 h. After standing for 2 h, the suspensions were then filtered through Whatman No. 42 filter paper. The soluble Cu(II) concentration was analyzed by AAS at 324.7 nm, and the amount of adsorbed Cu(II) was calculated from the mass difference in solution. Each sorption experiment was performed in triplicate.

#### 3.2.4. Statistical Analysis

All statistical analyses were performed using SPSS 21.0 software (SPSS Inc., Chicago, IL, USA).

## 4. Conclusions

A novel and effective low-cost adsorbent (the NMBCs) was successfully synthesized by precipitation. The NMBCs had a much stronger adsorption capacity (142 mg/g) for Cu(II) than did BC or NMnO_2_. The Cu(II) sorption process was described by a pseudo-second-order model. The Cu(II) adsorption capacity of the NMBCs was enhanced by increasing the pH, but was not affected by ionic strength. Thus, the Cu(II) adsorption on NMBCs was mainly due to the formation of surface complexes between Cu(II) and MnO_2_, as well as between Cu(II) and O-containing groups (such as O^2−^, C_2_H_3_O_2_–, and –OH). Owing to their high adsorption capacity and low cost, NMBCs may be an effective adsorbent for various environmental applications, such as wastewater treatment or the remediation of copper-contaminated soils.

## Figures and Tables

**Table 1:** Selected physiochemical properties of the BC, NMnO_2_, and NMBCs.

Sample	Bulk Elemental Composition (%)				Surface Atomic Composition (%)			Ash Content (%)	*S*_BET_ (m^2^/g)	Pore Width (nm)	*V*_tot_ (cm^3^/g)	pH_ZPC_
	C	H	O	N	C	O	Mn					
BC	85.3	5.2	5.16	0.81	75.0	15.3	-	10.2	61.0	23	0.036	10.0
NMnO_2_	-	-	-	-	17.48	45.38	31.91	-	161	2.54	0.020	7.80
NMBCs	73.4	2.1	17.2	0.68	35.6	41.2	19.68	12.6	80.3	3.86	0.013	11.0

**Table 2:** Parameters of the pseudo-first-order and pseudo-second-order models for the adsorption of Cu(II) by NMBCs and NMnO_2_.

Samples	Pseudo-First-Order			Pseudo-Second-Order		
	*q_e_*	*K*_1_	*R*^2^	*q_e_*	*K*_2_	*R*^2^
NMBCs	105.01	0.2	0.52	110.86	12.26	0.99
NMnO_2_	76.10	0.22	0.55	75.99	0.025	0.99

**Table 3:** Cu(II) adsorption isotherm parameters based on the Freundlich and Langmuir models.

Samples	Freundlich			Langmuir		
	*K*_F_ (mg^1−n^·L^n^/g)	*n*	*R*^2^	*q*_m_ (mg/g)	*b* (L/mg)	*R*^2^
BC	23.77 (0.35)	3.40 (0.06)	0.923	26.88 (0.23)	0.57 (0.07)	0.984
NMnO_2_	298.7 (0.27)	26.96 (0.11)	0.971	93.91 (0.22)	0.57 (0.05)	0.954
NMBCs	8316.6 (0.36)	632.91 (0.02)	0.974	142.02 (0.31)	0.81 (0.02)	0.978

**Table 4:** Thermodynamic parameters for the adsorption of NMBCs to Cu(II) at various temperatures.

*T* (K)	*q*_e_ (mg/g)	ln *K_e_*	Δ*G* (kJ/mol)	Δ*H* (kJ/mol)	Δ*S* (kJ/K·mol)	*R*
288	139.95	4.35	−10.41	0.45	0.038	0.980
298	142.02	4.32	−10.71	0.45	0.037	0.985
308	144.45	4.36	−11.16	0.45	0.038	0.983
